# Bottlebrush Elastomers with Crystallizable Side Chains: Monolayer-like Structure of Backbones Segregated in Intercrystalline Regions

**DOI:** 10.3390/polym16020296

**Published:** 2024-01-22

**Authors:** Evgeniia A. Nikitina, Erfan Dashtimoghadam, Sergei S. Sheiko, Dimitri A. Ivanov

**Affiliations:** 1 Faculty of Chemistry, Lomonosov Moscow State University (MSU), GSP-1, 1-3 Leninskiye Gory, 119991 Moscow, Russia; 2 Department of Chemistry, University of North Carolina at Chapel Hill, Chapel Hill, NC 27599-3290, USA; 3 Scientific Center for Genetics and Life Sciences, Sirius University of Science and Technology, 1 Olympic Ave, 354340 Sochi, Russia; 4 Institut de Sciences des Matériaux de Mulhouse-IS2M, CNRS UMR 7361, F-68057 Mulhouse, France

**Keywords:** bottlebrush elastomers, X-ray scattering, polymer crystallization

## Abstract

Bottlebrush (BB) elastomers with water-soluble side chains and tissue-mimetic mechanical properties are promising for biomedical applications like tissue implants and drug depots. This work investigates the microstructure and phase transitions of BB elastomers with crystallizable polyethylene oxide (PEO) side chains by real-time synchrotron X-ray scattering. In the melt, the elastomers exhibit the characteristic BB peak corresponding to the backbone-to-backbone correlation. This peak is a distinct feature of BB systems and is observable in small- or medium-angle X-ray scattering curves. In the systems studied, the position of the BB peak ranges from 3.6 to 4.8 nm in BB elastomers. This variation is associated with the degree of polymerization of the polyethylene oxide (PEO) side chains, which ranges from 19 to 40. Upon crystallization of the side chains, the intensity of the peak decays linearly with crystallinity and eventually vanishes due to BB packing disordering within intercrystalline amorphous gaps. This behavior of the bottlebrush peak differs from an earlier study of BBs with poly(ε-caprolactone) side chains, explained by stronger backbone confinement in the case of PEO, a high-crystallinity polymer. Microstructural models based on 1D SAXS correlation function analysis suggest crystalline lamellae of PEO side chains separated by amorphous gaps of monolayer-like BB backbones.

## 1. Introduction

In semicrystalline polymers, intercrystalline amorphous regions significantly impact several important properties, such as tensile strength [1], glass transition temperature [2,3], thermal expansion [3,4], and barrier properties [5], among others. Flory emphasized the significance of these regions, remarking, “The spatial form of polymer chains in the amorphous state (including intercrystalline domains) must be understood if we are to comprehend properties of polymers in molecular terms” [6]. Yet, directly assessing the structure of these intercrystalline domains presents a difficult experimental challenge. Consequently, scientists primarily rely on indirect methods for the characterization of amorphous chains. This includes, for instance, the utilization of dynamic mechanical analysis [7] to measure polymer relaxation parameters or the examination of miscibility behavior at order–disorder interphases.

The bottlebrush (BB) polymers are unique systems that exhibit the presence of the so-called “bottlebrush peak” in the small- or medium-angle X-ray scattering curves, which corresponds to the average spacing between the backbones [8,9]. This originates from the electron density contrast between the BB backbone and its side-chain shell. The observation of interchain correlation is a very particular characteristic of BB systems (melts, elastomers, gels) [10,11] distinct from their linear counterparts. This structural feature recently allowed for the visualization of the change in the chain conformation accompanying the crystallization of the BB elastomers with crystallizable poly(ε-caprolactone) side chains for the first time [12]. It was observed that, upon crystallization, the bottlebrush peak broadened and shifted to larger q values, attributed to the segregation of the BB backbones to the interlamellar regions, while parts of the side chains were progressively incorporated into the growing crystalline phase [12]. Therefore, for these systems, the time-resolved X-ray scattering provides the means not only to address the properties of the crystalline phase but also to probe packing of the amorphous chains confined in several nanometer-wide intercrystalline gaps.

Generally, (BB) polymers and copolymers have been garnering constant interest as a versatile platform for fabricating self-assembled materials with tissue-mimetic mechanical properties [11,13], holding promise for biomedical devices [14,15], pressure-sensitive adhesives [16], and organic electronics [17]. The unique properties of BB systems owe to molecular packing characterized by the architecturally suppressed overlap and entanglement of polymer chains [18,19,20]. Specifically, molecular BBs demonstrate disentangled polymer chains in the melt state, leading to a dramatic reduction in the rubber–elastic plateau modulus. Simultaneously, BB elastomers reveal significant stiffening during deformation. This combination of initial softness and intense strain-stiffening is vital for designing biomimetic materials capable of replicating the mechanical properties of soft living tissues. By introducing crystallizable side chains into such systems, another tuning mechanism becomes available, rendering these materials temperature-sensitive [21]. Fine-tuning the melting temperature of crystals built from BB side chains can precisely match, for instance, the body temperature. Such tailored thermal sensitivity can significantly facilitate the implantation of BB-based medical devices in the form of microneedles, where the materials, stiff at ambient temperature, undergo softening upon contact with the body to match the modulus of the surrounding tissue.

In this present work, we investigated the crystallization behavior of BB elastomers with polyethylene oxide side chains (PEO). Replacing PCL with PEO in BB side chains makes the resulting BBs water-soluble, opening doors for new applications [11,22]. However, the crystallization behavior of the BBs with these two oligomers in the side chain should differ significantly, especially concerning the final crystallinity values, as PEO belongs to the group of high-crystallinity polymers [23]. Therefore, we conducted a detailed in situ investigation of the crystallization behavior of the newly synthesized BBs, focusing on the evolution of the backbone configuration and the microstructure of the semicrystalline state.

## 2. Materials and Methods

**Scheme 1:**
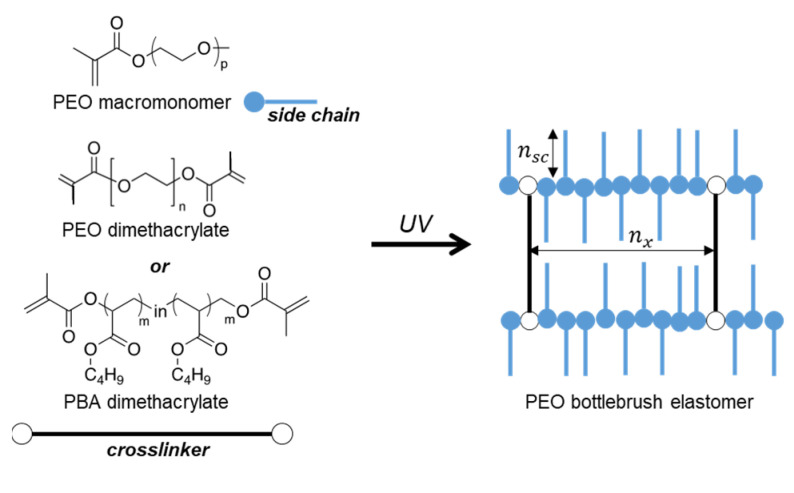
Synthesis of the BB elastomers from a PEO macromonomer using either a PEO crosslinker with Mn~6000 or a PBA-based crosslinker.

To investigate the effect of brush structure on crystallization, we synthesized two series of BB elastomers with different degrees of polymerization (DP) of PEO side chains ($n_{sc}$) and varied the DP of the backbone between crosslinks ($n_{x}$) (Table 1). The route employed for the fabrication of the BB elastomers is depicted in Scheme 1, while all synthetic details along with molecular characterization are provided in the Supporting Information.

The small- and wide-angle X-ray scattering (SAXS and WAXS) experiments were carried out at the BM26 beamline of the European Synchrotron Radiation Facility (ESRF) in Grenoble (France). The measurements were conducted in transmission geometry using photon energy of 12 keV. The accessed q values, with |q| = 4π sin(θ)/λ, where θ is the Bragg angle and λ is the wavelength, cover a range from 8.0 × 10^−2^ nm^−1^ to 4.0 nm^−1^. A Pilatus 1M detector (169 mm × 179 mm active area) was employed for recording SAXS intensity at a sample-to-detector distance of 3 m. WAXS patterns were collected simultaneously using a 300 K Pilatus detector (254 mm × 33.5 mm active area). In the experiments, the sample temperature was controlled with a THMS600 Linkam heating/cooling stage. The samples were preliminarily molten during short-term annealing at 80 °C, after which, they were cooled to −40 °C and then heated to 80 °C one more time at a rate of 12 K/min. The isothermal crystallization experiments were carried out upon melting at 80 °C and further fast cooling to the temperature of isotherm. The details about data reduction are provided in the Supporting Information.

The thermal behavior of the samples was investigated with a DSC 3+ (Mettler Toledo, Greifensee, Switzerland) differential scanning calorimeter using standard (10 °C/min) heating and cooling rates. Thermal programs and DSC curves for dynamic melting and crystallization are shown in Figure S1. The ∆H_m_ (melting enthalpy), T_m_, and T_c_ (melting and crystallization temperatures) were determined as the area and onset of the respective Gaussian peak.

## 3. Results

The semicrystalline structure of PEO bottlebrush elastomers depends on side chain length (${\sim n}_{sc}$) and crosslink density (${\sim n}_{x}$). Specifically, the melting temperatures are higher for the samples with $n_{sc}=40$, indicating that the corresponding crystals are thicker. On the other hand, for the same $n_{sc}$-values, the enthalpies of melting slightly decrease with the decrease in $n_{x}$, which can be attributed to the impact of crystal defects introduced by the crosslinks. The crystallization temperatures of all the BBs with the PEG crosslinker are rather close to each other. By contrast, the samples with the PBA crosslinker demonstrate significantly lower crystallization temperatures.

**Figure 1:**
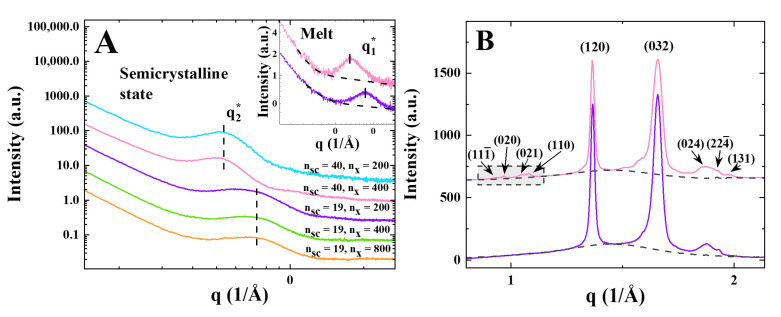
(**A**) SAXS curves of PGX_2k_200 (blue), PGX_2k_400 (pink), PGX_950_200 (purple), PGX_950_400 (green), and PGX_950_800 (orange) measured at −40 °C. Inset: Bottlebrush peak ex hibits low-q shift upon increasing side chain length from PGX_950_200 ($n_{sc}=19$, purple) to PGX_2k_400 ($n_{sc}=40$, pink) in the melt state. (**B**) WAXS curves plotted using the same color code as in panel A. For simplicity, the (032) peak indicates a superposition of overlapping $\left(13\bar{2}\right),\;\left(112\right),\;\left(21\bar{2}\right),\left(12\bar{4}\right),\;\left(20\bar{4}\right),$ and (004) reflections.

The small- and wide-angle X-ray scattering profiles of the samples (SAXS and WAXS) measured after cooling them from 80 °C (melt) to −40 °C at a rate of 12 K/min are given in Figure 1. In the melt, the elastomers exhibit the characteristic bottlebrush peak at $q_{1}^{*}$ (Figure 1A, inset). The peak positions are expected to exhibit scaling in accordance with $n_{sc}^{-3/8}$, aligning with previous studies of PCL brushes [12]. However, based on the two measurement points available, we cannot conclusively affirm this scaling in the present case. Upon crystallization, the semicrystalline structure is formed, with the appearance of a characteristic interference peak $q_{2}^{*}$ (the so-called long period $L_{p}$) in the SAXS region and sharp crystalline reflections in WAXS (Figure 1). The samples with longer side chains ($n_{sc}=40$) generate the $q_{2}^{*}$-peak at smaller q values (Figure 1A, inset), which is attributed to the formation of thicker crystals. Importantly, the bottlebrush peak is not visible for the semicrystalline state of all PEO samples (Figure 1A). This is in contrast to what has been previously observed for PCL brushes [12], where the evolution of the bottlebrush peak during crystallization was suggested as a marker for monitoring changes in the backbone configuration. 

**Figure 2:**
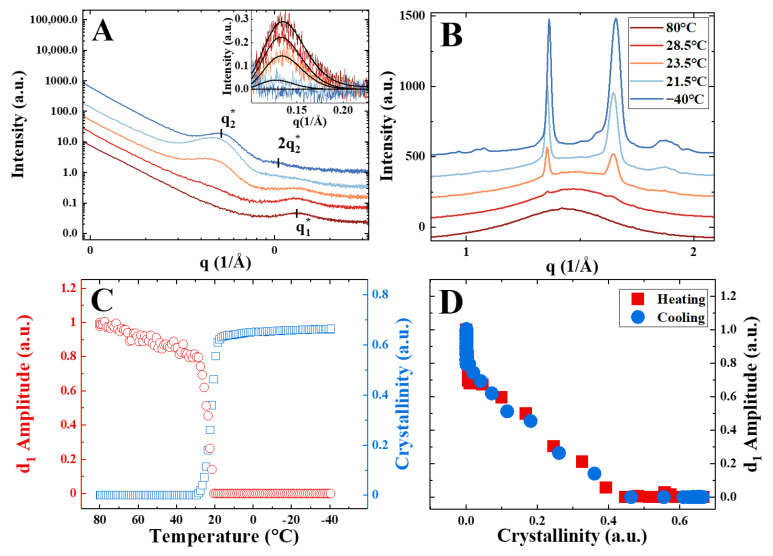
(**A**) Selected SAXS curves recorded during cooling from 80 °C to −40 °C at a rate of 12 K/min for sample PGX_2k_400; inset: bottlebrush peaks of the corresponding curves after background subtraction. (**B**) Corresponding WAXS curves with the temperature color code identical for panels A and B. (**C**) Evolution of WAXS crystallinity and amplitude of the bottlebrush peak during the cooling ramp. (**D**) Correlation of the amplitude of the bottlebrush peak and crystallinity for the cooling ramp (blue symbols) and for the subsequent heating ramp to 80 °C at the same rate (red symbols).

The exemplified WAXS curves presented in Figure 2B do not reveal major differences between the samples with long and short side chains. However, it should be noted that the samples with shorter side chains do not exhibit a group of weak WAXS peaks in the boxed q-region around 1.0 Å^−1^. This suggests a higher concentration of structural defects for the crystals with shorter chains. The microstructural parameters of the semicrystalline structures are summarized in Table 2.

The crystal thickness from SAXS shows that the BBs with longer side chains form thicker crystals, which is consistent with the DSC measurements (Table 1). This increase is accompanied by the thickness increase in the interlamellar amorphous regions $L_{a}$.

To better understand the BB packing transformations during crystallization and melting, in situ dynamic and isothermal crystallization experiments were performed using the synchrotron source. The synchrotron heating/cooling experiments are exemplified in Figure 2 for the case of sample PGX_2k_400. The SAXS-WAXS curves recorded during crystallization show the steadily growing interference peak concurrently with the increasing intensity of the WAXS reflections.

In contrast, the bottlebrush peak progressively vanishes (Figure 2A, inset), leaving the place for the second order of the interference peak. To appreciate the simultaneous decay of the bottlebrush peak and increase in WAXS crystallinity, the temperature dependencies of the two parameters are presented in Figure 2C. The initial decrease in the $d_{1}$-amplitude in the temperature range from 80 to 30 °C is attributed to the change in the electron density contrast between the phases of the phase-separated morphology due to thermal contraction.

Starting from the onset of crystallization, the decrease in the $d_{1}$-amplitude becomes sharper and reaches zero much before the completion of crystallization (Figure 2D). The heating and cooling traces completely superimpose, suggesting that the bottlebrush peak behavior is largely determined by the sample crystallinity. In the crystallinity range between zero and ~0.45, the dependence of the $d_{1}$-amplitude on crystallinity is linear. This signifies that the consumption of the bulk amorphous phase results in the progressive disappearance of the bottlebrush peak, which is completely absent from the curves in the final semicrystalline state.

## 4. Discussion

The obtained data are in contrast to the previously studied PCL brushes [12]. The fact that the bottlebrush peak completely vanishes is explained by a significantly smaller thickness of the amorphous gaps for this high-crystallinity polymer compared to PCL. In the PCL brushes, ($n_{sc}=$ 13), $L_{a}$ = 9 nm is notably larger than the d-spacing of the corresponding $d_{1}$-peak of the unperturbed bulk amorphous phase (in the melt, the BB diameter $d_{1}$ = 4.9 nm). In contrast, the intercrystalline amorphous gap thickness for the PGX_2k_400 sample in the semicrystalline state ($L_{a}$ = 3.6 nm) is significantly smaller than the BB diameter in the melt ($d_{1}$ = 4.8 nm). This means that crystallization-induced confinement of BB backbones in PEG bottlebrushes is much stronger.

The rejection of the BBs to intercrystalline gaps and the incorporation of significant parts of the side chains in the growing crystals completely destroy the initial packing of the BBs in the unperturbed amorphous phase. This makes the observation of the backbone-to-backbone correlation in a semicrystalline state of PEO bottlebrushes impossible. The fact that the bottlebrush peak disappears prior to the end of crystallization suggests the imperfect packing of lamellar stacks, where the lamellae growth in the amorphous gaps between the lamellar stacks dominates when crystallinity exceeds 0.45. Therefore, the intensity of the bottlebrush peak provides a direct measure of the consumption of the unperturbed bulk amorphous phase in the course of crystallization.

**Figure 3:**
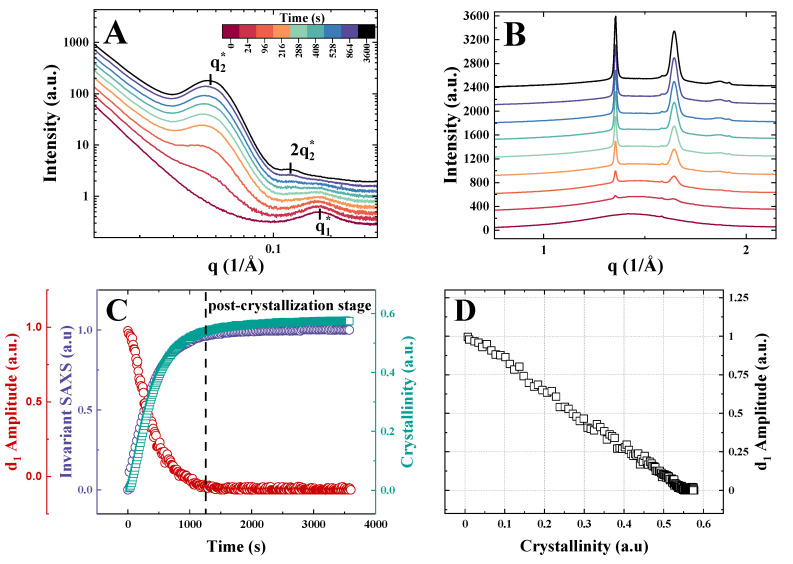
(**A**) Selected SAXS curves recorded during isothermal melt crystallization of sample PGX_950_200 at 24 °C; the time scale is given in color code. (**B**) Corresponding WAXS curves with the same color code. (**C**) Time evolution of the WAXS crystallinity index, amplitude of the bottlebrush peak, and SAXS invariant. (**D**) Dependence of d_1_-amplitude on the WAXS crystallinity index.

The isothermal crystallization of PEO brushes is illustrated using PGX_950_200 as an example (Figure 3). The SAXS-WAXS curves confirm the above-discussed general trends during cooling and heating ramps. Similarly to the nonisothermal crystallization regime, the bottlebrush peak vanishes during isothermal crystallization. Yet, the SAXS curves reveal less variation in the main interference peak position, which is normal for the isothermal crystallization. In Figure 3C, the bottlebrush peak amplitude is plotted together with the WAXS crystallinity index and SAXS invariant. Like in the case of the nonisothermal regime, the correlation of the $d_{1}$-amplitude and WAXS crystallinity index is linear, with zeroing of the bottlebrush peak intensity occurring slightly above 0.5. The small difference in the cut-off crystallinity between the two crystallization conditions may be linked to more regular lamellar stacking during isothermal crystallization, which would leave less interstack amorphous gaps upon the impingement of banded spherulites (not shown here). On this figure panel, a post-crystallization stage is specifically marked. This stage corresponds to the situation when the amplitude of the bottlebrush peak has already reached zero levels, indicating that the bulk amorphous phase is fully consumed, and the SAXS invariant has leveled out at its maximum values. However, the WAXS crystallinity still shows a small additional increment due to crystal growth in confined spaces. This observation is in line with what has been found for the isothermal PCL brush crystallization [13], also exhibiting a post-crystallization stage.

**Figure 4:**
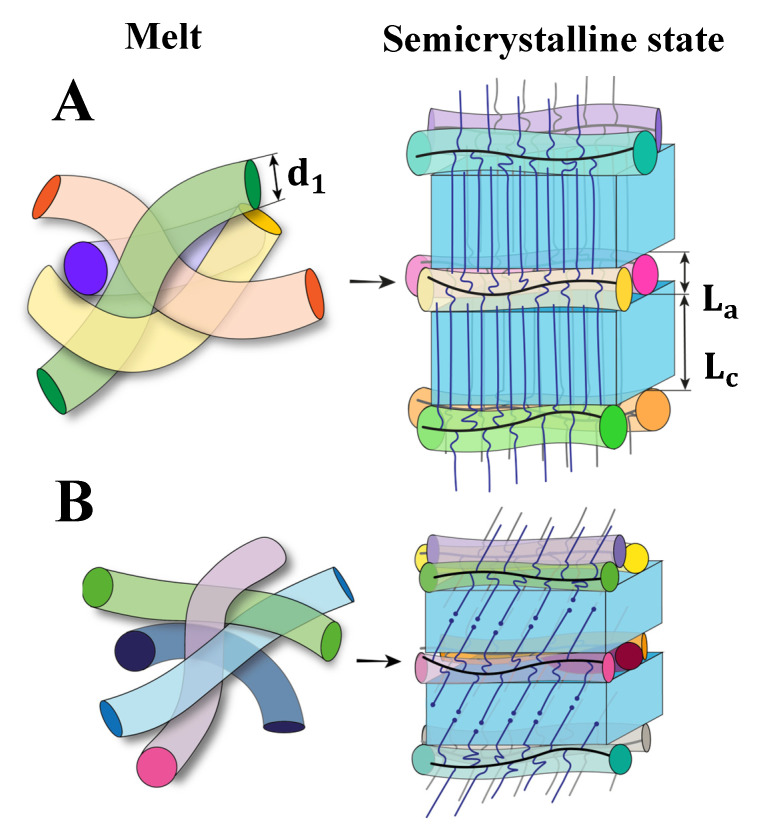
Schematics of the backbone and side-chain arrangement in the molten and semicrystalline states of brush elastomers with $n_{sc}$ = 40 (**A**) and $n_{sc}$ = 19 (**B**).

The configuration of the side chains and main chain in the semicrystalline state can be deduced from the comparison of the SAXS crystal thickness and side chain length. For $n_{sc}\;$ = 40, the $L_{c}$-values range between 7.5 and 7.9 nm, which correspond to 27–28 monomers for the classical (7/2) helical conformation of PEO [22]. In this case, the backbone with the remaining $n_{a}=n_{sc}-L_{c}*\frac{7}{c}=$12–13 side-chain monomers are incorporated in the interlamellar amorphous region. This amorphous part of BB molecules serves as a fold connecting the crystalline stems in different crystallographic positions. It is known from the literature that the sharp regular folds on the surface of solution-grown PEO crystals can be as short as 3.5 monomers [24], suggesting that crystallization leaves enough chain length for the formation of diverse folds, including adjacent reentry folds, as well as more loose folds. The latter can involve sequences of several PEO monomers located close to the backbone. Assuming that the PEO stems are not inclined with respect to the basal (b*c*) lamellae plane, the amorphous part of the bottlebrush should fit into the interlamelar gap of thickness $L_{a}$. Given that all crystallographic positions are filled with crystalline stems, the flux (Φ) of amorphous chains emanating from the crystal surface should have a total density of approx. 4.7 chain/nm^2^. This condition comes from the fact that the (b*c*) section of the unit cell accommodates four PEO chains. Therefore, the total height of the amorphous parts of the side chains protruding from the crystal surface can be estimated as follows:

(1) $$h=\frac{n_{a}M_{mon}}{N_{A}\rho_{a}}\Phi ,$$

where $M_{mon}$ is the PEO monomer mass, $\rho_{a}$ is the mass density of amorphous PEO, and $N_{A}$ is the Avogadro number. For simplicity, Equation (1) neglects the volume of the main-chain monomer as it is much smaller than the volume of $n_{a}$ side-chain monomers. Using $\rho_{a}$ = 1.12 g/cm^3^, (see, e.g., Ref. [25]) we calculate h ranging between 3.7 and 4.0 nm. These values are close to $L_{a}$, which means that the structure of the interlamellar amorphous gap contains approximately one monolayer of the bottlebrush backbones. The schematics of the semicrystalline structure are depicted in Figure 4A.

The situation is different for the samples with shorter side chains ($n_{sc}$ = 19). In this case, the lamellar thickness is comprised between 5.4 and 5.8 nm, which is equivalent to the crystalline stem length of 19–21 monomers. It is, therefore, clear that individual side chains cannot form stems in a simple way. One likely alternative would be the formation of the so-called “half-stemmed” crystal [26], where the stem length is about one half of a bilayer crystal thickness with chain ends entering the crystalline phase (Figure 4B). Although such a microstructure is unconventional, the general possibility of the PEO lattice to incorporate different chain defects is well known. For example, the PEO chains containing a 1,2,3-triazole ring in the central position of the chain (PEO_11_-TR-PEO_11_) can be incorporated in the crystalline lattice forming a composite bilayer crystal [27]. The presence of defects (chain ends) in PEG crystals of the BB with short side chains $n_{sc}=19$ can be inferred from the notable discrepancy between linear and bulk crystallinity, approximately 20%. In contrast, in crystals with longer chains ($n_{sc}=40$), this difference is limited to only a few percent, as indicated in Table 2. Additionally, the absence of WAXS peaks near q = 1.0 Å^−1^ (cf. Figure 2B), as mentioned earlier, suggests a higher concentration of structural defects in crystals with shorter chains.

Considering that in the bilayer structure, the h value should exhibit a two-fold increase, this results in h=5.2–5.8 nm. Since the experimentally observed $L_{a}^{\prime }s$ are much smaller, one should introduce an additional modification to the microstructure. This additional feature is likely the chain tilt in the crystal. Generally, it is known that chain tilts are typical for polymers forming planar zigzag conformations in the crystalline state and having a significant density difference between the crystalline and amorphous phases [28]. The nonplanar chain conformations are seemingly less concerned with such features [29] (see, e.g., the results of a direct chain tilt measurement for the bulk sample of polytrimethylene terephthalate [30]). However, there are reports on the occurrence of significant chain tilts in the crystals of cyclic PEO [31]. The studied architecture of the PEO brushes might also be prone to forming tilted stems due to topological problems at the crystal–amorphous interface related to the covalent bonding of the side chains to the main backbone. Assuming the tilt angle φ with respect to the lamellar is normal, the $n_{a}$ and h are calculated as follows:

(2) $$n_{a}=n_{sc}-L_{c}\times 7/(2c\times cos\varphi ),$$

(3) $$h=\frac{2n_{a}M_{mon}}{N_{A}\rho_{a}}\Phi cos\varphi .$$

The coefficient 2 in Equation (3) results from the bilayer structure, where chains in each amorphous region are covalently bound to both neighboring crystals instead of just one, as in the case of $n_{sc}$ = 40. By substituting Equation (2) into (3), one obtains the following:

(4) $$cos\varphi =\frac{N_{A}\rho_{a}h}{2M_{mon}\Phi n_{sc}}+\frac{{7L}_{c}}{2cn_{sc}}.$$

Replacing h with $L_{a}\prime s$ found from the SAXS correlation function analysis in Equation (4), we estimate φ≈ 43°. The corresponding schematics are shown in Figure 4B. It is noteworthy that direct measurements of the tilt angle would be necessary to verify the proposed molecular packing model.

Generally, the formation of half-stemmed crystals requires nuclei having crystalline stems spanning the entire lamellar thickness. The side-chain polydispersity, according to the supplier specification [32], makes occurrences of side chains with $n_{sc}$ = 17.0 ÷ 23.9 possible. Therefore, the formation of such nuclei cannot be precluded. In addition, the PEG crosslinks present in the system have molecular weights of ~6000 and can also serve as templates for the formation of such crystals. To appreciate the efficiency of nucleation on the crosslinks, it is instructive to compare the crystallization kinetics in the systems with PEG and PBA crosslinks. As noted above, the Tcs of both systems dramatically differ. An example of the crystallization of a PBA-containing sample is given in Figure S4 (Supporting Information). The crystallization of PBX_950_300 at 5 °C occurs approx. at the same rate as the crystallization of PGX_950_200 at 24 °C. This shows that the presence of a PEG linker significantly accelerates the process. Therefore, the polydispersity of side chains alone is probably insufficient to ensure the nucleation of such composite crystals.

## 5. Conclusions

In conclusion, the microstructure and phase transitions of bottlebrush elastomers containing PEO side chains with DPs of 19 and 40 were investigated with synchrotron X-ray scattering. It was observed that the characteristic bottlebrush peak with the d-spacing of 3.6–4.8 nm, which is present in the unperturbed bulk amorphous phase, progressively vanishes upon crystallization. This is explained by the rejection of the bottlebrush backbones from the growing crystals and their confinement within intercrystalline gaps of 2.1–3.6 nm. The complete disappearance of the bottlebrush peak is in contrast with previous studies on the bottlebrushes with poly(ε-caprolactone) side chains and can be accounted for by the higher crystallinity of PEO (52–67%) and stronger backbone confinement. The analysis of the microstructure of the semicrystalline state of the bottlebrushes suggests that the backbones form a layer with a thickness comparable to that of a monolayer. This configuration of the intercrystalline amorphous regions is in line with the high crystallinity of the final morphology.

From the perspective of using newly synthesized materials in biomedical applications, there may be a preference for bottlebrush elastomers with a degree of polymerization (DP) of 19 for the polyethylene oxide (PEO) side chains, as opposed to 40. This preference is due to the melting point of the corresponding crystals being closer to the human body temperature. As a result, materials based on these systems will soften upon insertion into the human body, making them more suitable for such applications.

## Figures and Tables

**Table 1:** Molecular and thermal characteristics of the synthesized BB elastomers.

Sample Name	n_sc_^1^	n_x_^2^	Cross-Linker	ΔH_m_^3^ J/g	T_m_^4^ °C	T_c_^4^ °C
PGX_950_200	19	200	PEO	79.6	35.2	9.3
PGX_950_400	19	400	PEO	81.1	35.9	8.5
PGX_950_800	19	800	PEO	85.2	38.4	6.9
PGX_2k_200	40	200	PEO	80.2	43.3	13.7
PGX_2k_400	40	400	PEO	87.3	43.4	14.9
PBX_950_150	19	150	PBA	69.1	30.1	−21.8
PBX_950_300	19	300	PBA	72.8	34.6	−20.9

^1^ Degree of polymerization (DP) of the PEO side chains; ^2^ DP of the backbone between crosslinks; ^3^ enthalpy of melting extracted from DSC measurements performed at a rate of 10 K/min; ^4^ melting and crystallization temperatures.

**Table 2:** Microstructural characteristics of the synthesized PEO bottlebrush networks at −40 °C.

Sample Name	d_1_^1^ nm	L_p_^2^ nm	L_a_^2^ nm	L_c_^2^ nm	$\alpha_{SAXS\;}$ ^3^	$\alpha_{WAXS\;}$ ^4^
PGX_950_200	3.6	8.0	2.6	5.4	67.5	52.6
PGX_950_400	3.6	8.0	2.3	5.7	71.3	53.9
PGX_950_800	3.6	7.9	2.1	5.8	73.4	52.2
PGX_2k_200	4.9	11.0	3.5	7.5	68.2	67.0
PGX_2k_400	4.8	11.5	3.6	7.9	68.7	66.5
PBX_950_150 ^5^	3.7	8.0	2.7	5.3	66.3	56.6
PBX_950_300 ^5^	3.7	7.2	2.3	4.8	67.6	56.0

^1^ d-spacing of the $q_{1}^{*}$-peak of the bottlebrush melt determined as ${2\pi /q}_{1}^{*}$; ^2^ long period (*L_p_*), crystal thickness (*L_c_*), and amorphous layer thickness (L_a_) determined from the 1D SAXS correlation function analysis; ^3^ SAXS linear crystallinity defined as $L_{c}/L_{p}$; ^4^ WAXS crystallinity index computed as an integral intensity of the crystalline WAXS peaks normalized by the total scattering intensity; ^5^ SAXS/WAXS data are given in Figure S3.

## Data Availability

Data are contained within the article.

## Supplementary Materials

The following supporting information can be downloaded at: https://www.mdpi.com/article/10.3390/polym16020296/s1, Figure S1: DSC traces of the synthesized PEO brushes, the macromonomer and PEG crosslinker measured on heating and cooling at a rate of 10 K/min; Figure S2: Left: 1D SAXS correlation functions used for calculation of thickness of the lamellar ($L_{c}$) and interlamellar amorphous layer ($L_{a}$) layer. Right: Example of decomposition of WAXS intensity (black) into crystalline peaks (blue) and amorphous halo (red) for sample PEG_2k_400; Figure S3: A: SAXS curves of samples PBX_950_150 and PBX_950_300 recorded at −40 °C after melting at 80 °C. B: WAXS curve corresponding to sample PBX_950_150; Figure S4: A: Selected SAXS curves recorded during isothermal melt crystallization of sample PBX_950_150 at 5 °C, the time scale is given in color code. B: Time evolution of the WAXS crystallinity index, amplitude of the bottlebrush peak and SAXS invariant. Inset: time evolution of the lamellar thickness; Table S1: Molecular and thermal characteristics of the macromonomer and PEO crosslinker; Table S2: Microstructural characteristics of PBX_950_150 after melt crystallization at 5 °C for 3000 s. References [33,34,35,36] are cited in the supplementary materials.
