# Supplementary material for: Bottlebrush Elastomers with Crystallizable Side Chains: Monolayer-like Structure of Backbones Segregated in Intercrystalline Regions
Source: Polymers (Basel). 2024 Jan 22;16(2):296. doi: 10.3390/polym16020296 (PMC10819367; doi:10.3390/polym16020296)
Supplement: Supplementary file 1 [file polymers-16-00296-s001.zip › polymers-2786380-supplementary.pdf]

Supporting Information

Bottlebrush Elastomers with Crystallizable Side Chains: Monolayer-Like Structure of Backbones Segregated in Intercrystalline Regions

Evgeniia A. Nikitina [1], Erfan Dashtimoghadam [2], Sergei S. Sheiko [2,*] and Dimitri A. Ivanov [1,3,4,*]

1. Faculty of Chemistry, Lomonosov Moscow State University (MSU), GSP-1, 1-3 Leninskiye Gory, 119991 Moscow, Russia
2. Department of Chemistry, University of North Carolina at Chapel Hill, Chapel Hill, NC 27599-3290, USA
3. Scientific Center for Genetics and Life Sciences, Sirius University of Science and Technology, 1 Olympic Ave, 354340 Sochi, Russia
4. Institut de Sciences des Matériaux de Mulhouse-IS2M, CNRS UMR 7361, F-68057 Mulhouse, France

* Correspondence: sergei@email.unc.edu (S.S.S.); dimitri.ivanov@uha.fr (D.A.I.)

1. Chemical Synthesis

Materials. Poly(ethylene oxide) (PEO) methyl ether methacrylate (M_n~950), PEO dimethacrylate (M_n~6,000), polyethylene glycol with a molecular weight of 2000 Da (PEG 2000), phenylbis(2,4,6-trimethylbenzoyl)phosphine oxide (BAPO), *n*-butyl acrylate (n-BA), ethylene bis(2-bromoisobutyrate) (2-BiB), copper(II) bromide ($CuBr_2$), tris[2-(dimethylamino)ethyl]amine (Me_6TREN), triethylamine, methacryloyl chloride, and anhydrous magnesium sulfate were purchased from Sigma-Aldrich. PEO methyl ether methacrylate (solution) and *n*-BA were purified by passing through a column of basic alumina (Sigma Aldrich) to remove inhibitor. Dichloromethane (DCM), acetonitrile, chloroform, N,N-dimethylacetamide, p-xylene, and tetrahydrofuran (THF) were used as received from Sigma Aldrich.

PEO 2000 methacrylation. Hydroxyl groups on PEO 2000 were methacrylated to synthesize macromonomers with a molecular weight of 2000 Da. 20 g PEO 2000 was dissolved in 100 mL DCM and dried with anhydrous magnesium sulfate overnight. The solution was filtered and transferred to a dry 200 mL flask. 0.8 mL Triethylamine was added to the flask and the mixture was cooled using an ice bath. Subsequently, 1.2 g methacryloyl chloride dissolved in 20 mL anhydrous DCM was added to the mixture dropwise. The ice bath was removed, the temperature was increased to room temperature, and the reaction was continued overnight. The mixture was filtered, macromonomers were purified, and fully dried.

Synthesis of poly(butyl acrylate) (PBA) macro-crosslinker. PBA macro-crosslinker was synthesized by atom transfer radical polymerization using a difunctional initiator (2-BiB). The macro-crosslinker ($n_{sc} = 80$) was synthesized by combining 24 g of n-BA, Me_6TREN (2 μL), $CuBr_2$ (1.6 mg), and 2-BiB (0.67 g) and diluting the mixture to 50% with acetonitrile. The reaction was then cooled in an ice bath and degassed for 1 hour with bubbling nitrogen gas (N_2). The polymerization was initiated by the addition of a Cu^0 wire and transferred to a 45°C oil bath until the reaction reached ~80% conversion. The reaction was then terminated by the addition of 50 mL of chloroform and washed with water. Solvent was removed by rotary evaporation at 45°C under reduced pressure. The cleaned polymer was then functionalized by the addition of 7 parts N,N-dimethylacetamide and a large excess of potassium methacrylate and left stirring for 72 hours. 50 mL of chloroform and 100 mL of water were then added separating the polymer into the organic phase. The organic phase was then washed in water until it became clear. Solvent was again removed by rotary evaporation at 45°C under reduced pressure.

Synthesis of PEO bottlebrush elastomers. Poly(ethylene oxide) (PEO) bottlebrush elastomers were prepared by one-step polymerization of PEO methyl ether methacrylate macromonomers (M_n~950 and 2,000 g/mol, Sigma-Aldrich) with different molar ratios of PEO dimethacrylate (M_n~6,000) or poly(butyl acrylate) macro-crosslinker. The initial reaction mixtures contained 50 wt.% macromonomers, different molar ratios of macro-crosslinker, 1.5 wt.% BAPOs photoinitiator, and p-xylene as solvent. First, the mixtures were degassed by N_2 bubbling for 30 minutes. Subsequently, to prepare films, the mixtures were injected between two glass plates with a 2.3 mm spacer and polymerized at room temperature for 12 hrs under N2. Films were then washed with THF (two times with enough to immerse and fully swell the films, each time for 8 hrs) in glass Petri dishes. The samples were finally dried at room temperature. The conversion of monomers to elastomers (gel fraction) was between 90 to 98 wt.% in every case.

2. Thermal Characterization of the PEO Bottlebrushes

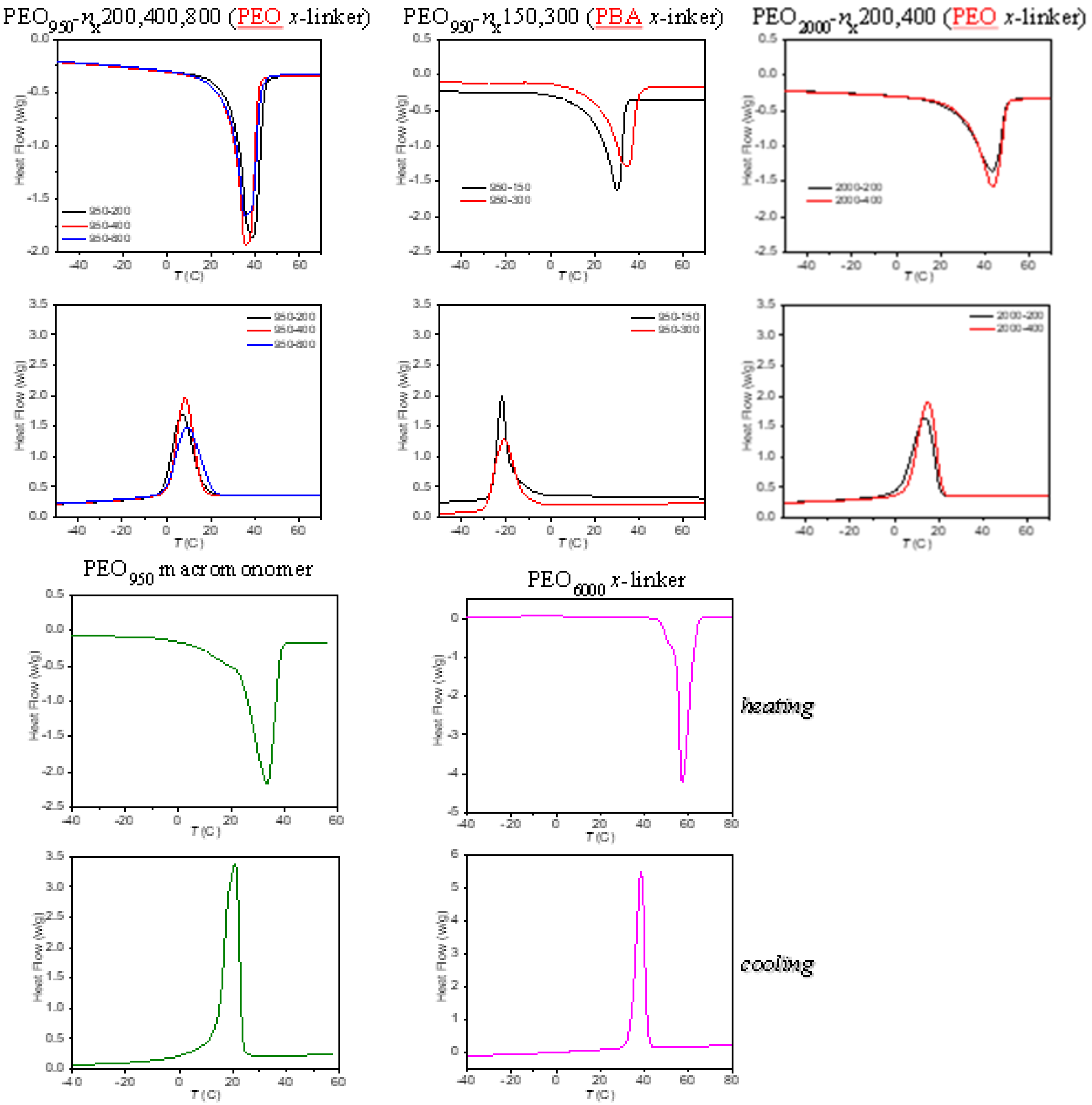

Figure S1: DSC traces of the synthesized PEO brushes, the macromonomer and PEG crosslinker measured on heating and cooling at a rate of 10 K/min.

Table S1: Molecular and thermal characteristics of the macromonomer and PEO crosslinker

Sample	side-chain (M_n ~)	n_{sc}	n_x	x-linker	T_m (°C)	ΔH_m (J/g)	T_c (°C)	ΔH_c (J/g)
Macromonomer	950	19	--	--	33.2	114.7	20.8	114.2
PEO crosslinker	6000	--	--	--	57.3	158.6	38.4	159.3

3. X-ray Scattering Experiments

The 1D data reduction, correction and calibration was performed using the PyFAI package described elsewhere.[33] For quantitative analysis of SAXS curves the 1D correlation and interface distribution functions (IDF) were calculated as described previously.[34,35] To compute the WAXS crystallinity index, the experimental curves were fit to a sum of Gaussian functions representing each of the crystalline reflexes of the PEG lattice plus a broad peak (amorphous halo) modelled using Lorentzian function. The crystallinity index was computed as a ratio of the surface under the crystalline reflexes to the total scattering intensity.[36]

After correction for the empty cell, the background intensity was modelled with a double exponential function $f(q) = I_0 + A_1 e^{-(q-q_0)/t_1} + A_2 e^{-(q-q_0)/t_2}$.
The SAXS invariant was calculated by integrating the intensity of the fully corrected SAXS curves up to the q-value of 2 nm^{-1}. The data modelling and analysis were performed using custom-built routines designed in the Igor Pro environment (WaveMetrics Ltd.) and scripts written using scientific Python libraries.

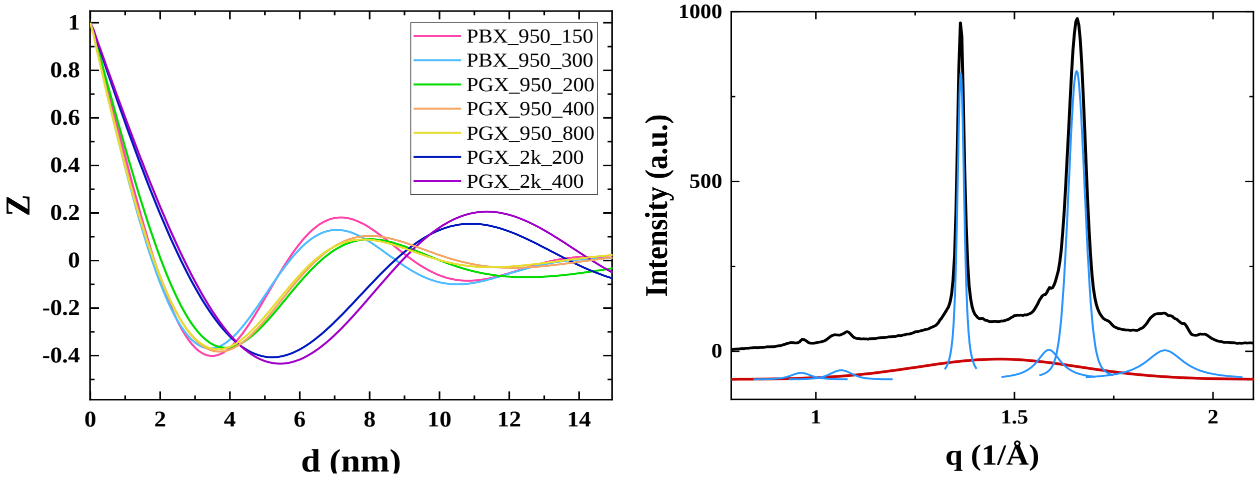

Figure S2: *Left*: 1D SAXS correlation functions used for calculation of thickness of the lamellar (L_c) and interlamellar amorphous layer (L_a) layer. *Right*: Example of decomposition of WAXS intensity (black) into crystalline peaks (blue) and amorphous halo (red) for sample PEG_2k_400.

4. SAXS/WAXS Curves of PBX Samples

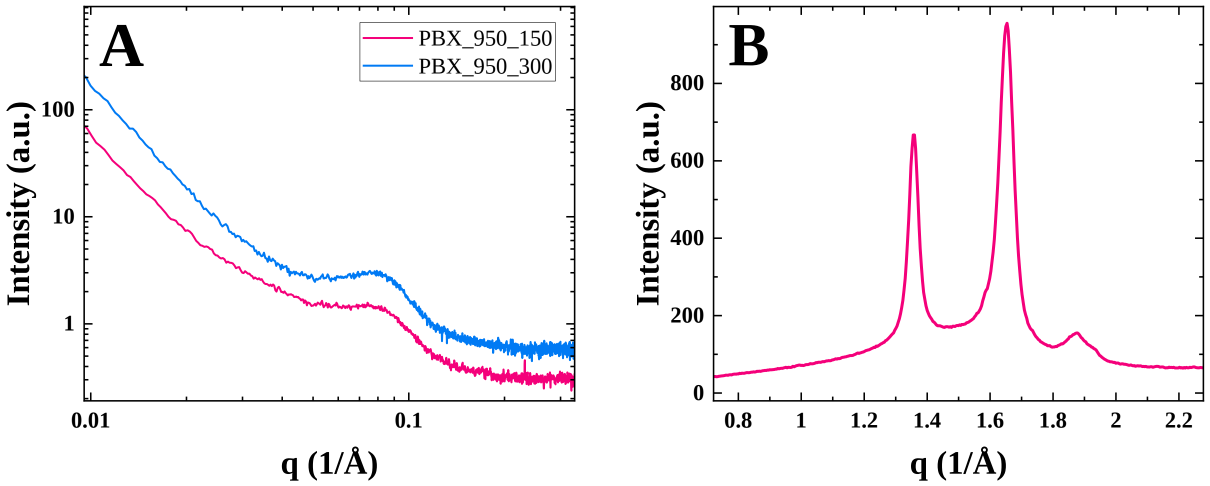

Figure S3: A: SAXS curves of samples PBX_950_150 and PBX_950_300 recorded at -40 °C after melting at 80°C. B: WAXS curve corresponding to sample PBX_950_150.

5. Isothermal Crystallization of PBX_950_300 at 5°C

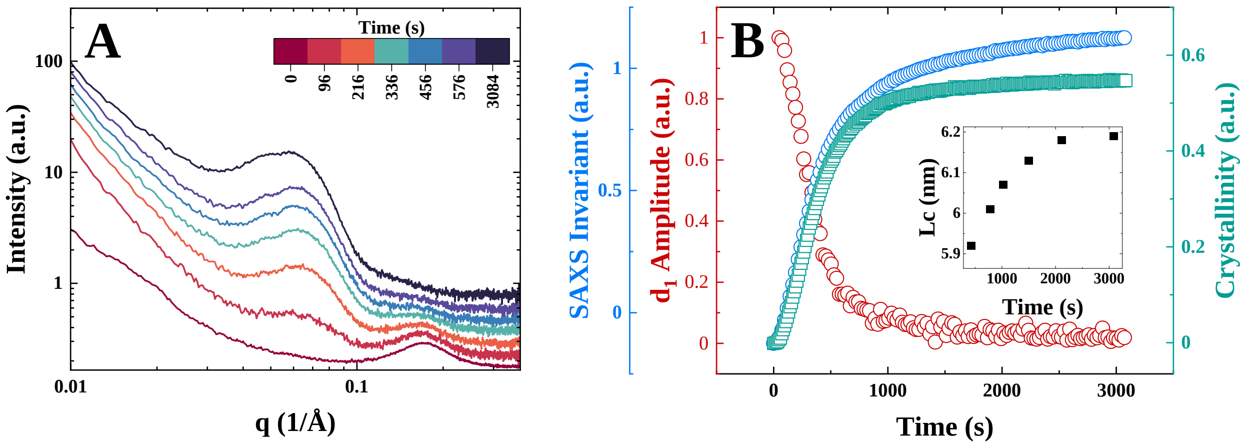

Figure S4: *A*: Selected SAXS curves recorded during isothermal melt crystallization of sample PBX_950_150 at 5 °C, the time scale is given in color code. *B*: Time evolution of the WAXS crystallinity index, amplitude of the bottlebrush peak and SAXS invariant. Inset: time evolution of the lamellar thickness.

Table S2: Microstructural characteristics of PBX_950_150 after melt crystallization at 5 °C for 3000 s.

L_p, nm	9.6
L_c, nm	6.2
L_a, nm	3.1
Crystallinity (SAXS), %	66.6
Crystallinity (WAXS), %	54.7
